# Gamma-Polyglutamic Acid-Rich Natto Suppresses Postprandial Blood Glucose Response in the Early Phase after Meals: A Randomized Crossover Study

**DOI:** 10.3390/nu12082374

**Published:** 2020-08-08

**Authors:** Risa Araki, Takeshi Yamada, Kazushi Maruo, Akihiro Araki, Rena Miyakawa, Hiroaki Suzuki, Koichi Hashimoto

**Affiliations:** 1 Department of Clinical and Translational Research Methodology, Faculty of Medicine, University of Tsukuba, 1-1-1 Tennodai, Ibaraki, Tsukuba 305-8575, Japan; risa.araki@md.tsukuba.ac.jp (R.A.); s1921307@s.tsukuba.ac.jp (R.M.); 2 Food Research Institute of National Agriculture and Food Research Organization, 2-1-12 Kannondai, Ibaraki, Tsukuba 305-8642, Japan; 3 R&D Center for Tailor-Made QOL, University of Tsukuba, 1-2 Kasuga, Ibaraki, Tsukuba 305-8550, Japan; 4 AIST-University of Tsukuba Open Innovation Laboratory for Food and Medicinal Resource Engineering (FoodMed-OIL) 1-1-1 Higashi, Ibaraki, Tsukuba 305-8565, Japan; 5 Department of Gastroenterology, Faculty of Medicine, University of Tsukuba, 1-1-1 Tennodai, Ibaraki, Tsukuba 305-8575, Japan; t.yamada718@md.tsukuba.ac.jp; 6 Department of Biostatistics, Faculty of Medicine, University of Tsukuba, 1-1-1 Tennodai, Ibaraki, Tsukuba 305-8575, Japan; maruo@md.tsukuba.ac.jp; 7 Faculty of Health Science, Tsukuba International University, 6-8-33 Manabe, Ibaraki, Tsuchiura 300-0051, Japan; a-araki@tius.ac.jp; 8 Graduate School of Comprehensive Human Sciences, University of Tsukuba, 1-1-1 Tennodai, Ibaraki, Tsukuba 305-8575, Japan; 9 Department of Internal Medicine (Endocrinology and Metabolism), Faculty of Medicine, University of Tsukuba, 1-1-1 Tennodai, Ibaraki, Tsukuba 305-8575, Japan; hirosuzu@md.tsukuba.ac.jp

**Keywords:** gamma-polyglutamic acid, natto, postprandial glucose, meal loading test, human health

## Abstract

We evaluated the suppressive effects of high-gamma-polyglutamic acid (*γ*-PGA) natto on postprandial blood glucose level and insulin response. After confirming the eligibility of candidates using a pre-selective test with packaged white rice, a meal loading test including low- or high-*γ*-PGA natto (with 57.6 mg (LPGA) and 439.6 mg (HPGA) of *γ*-PGA, respectively) was conducted in men aged 20 to 70 years (*n* = 29) and postmenopausal women aged ≤70 years (*n* = 7). On each examination day, blood samples were obtained after they fasted overnight and for 120 min after test meal loading. The primary outcome of this study was the difference between the measurements of the incremental area under the curve (IAUC) for blood glucose 0 to 30 min after loading of LPGA and HPGA meals. The IAUCs for blood glucose and insulin after the HPGA meal were lower than those after the LPGA meal within 45 min (0 to 15 and 0 to 30 min: *p* < 0.001, 0 to 45 min: *p* < 0.01) and 1 h (all *p* < 0.001) of loading, respectively. The suppressive effects of HPGA natto on postprandial glucose response in the early phase, which possibly relates to the risk of dysglycemia and cardiovascular disease, were clarified.

## 1. Introduction

Recently, the use of functional ingredients in agricultural products to prevent lifestyle-related diseases has gained attention [1]. Natto is a traditional Japanese food made from soy through fermentation by *Bacillus subtilis* and is rich in soybean protein, isoflavone, dietary fiber, and vitamin K, which are derived from soy and produced by *Bacillus subtilis* [2]. The gamma-polyglutamic acid (*γ*-PGA) is a viscous substance produced during the fermentation of natto [3]. The high-*γ*-PGA (HPGA) natto showed higher viscosity scores than low-*γ*-PGA (LPGA) natto in sensory evaluation tests and possible effectiveness of HPGA natto in inhibiting the elevation of postprandial blood glucose within 1 h after a meal was observed in our previous study [4]. β-glucan, a water-soluble dietary fiber, is a highly viscous substance [5] similar to *γ*-PGA. Higa et al. reported that postprandial glucose levels are suppressed in response to a mixture of high-β-glucan barley with white rice (WR) 30 min after test meal loading in healthy participants [6]. Ames et al. compared the postprandial glucose levels after loading with tortillas prepared from flours with various β-glucan contents and revealed a greater decrease in the percent change in glucose levels from the baseline to 30 min after high-β-glucan treatments than that observed in response to low- and medium-β-glucan treatments [7]. It has also been reported that the concomitant intake of Mekabu, a seaweed rich in viscous fiber, lowers the incremental area under the curve (IAUC) of blood glucose 30 min after meal loading [8]. Therefore, we expected that the viscous properties of foods suppress postprandial glycemic responses, and the HPGA natto with higher viscosity would be more effective than LPGA natto with lower viscosity. Furthermore, we considered the suppression of plasma glucose elevation from the baseline to 30 min after a meal could be a relevant time period for evaluating the effects of HPGA natto in humans. Although not results from an interventional study, Peddinti et al. reported that the 1 h and 30 min postprandial glucose levels are effective biomarkers of dysglycemia [9]. Individuals with dysglycemia, especially postprandial hyperglycemia, are at high risk of cardiovascular disease [10]. In addition, in an interventional study, the peak blood glucose level within 1 h post-loading was considered to be related to the intima-media thickness of the carotid artery (CIMT) [11]. However, our previous exploratory study [4] was designed to focus on blood glucose elevations 2 h after eating and had some limitations, such as small sample size, the inclusion of individuals exhibiting a small range of glycemic fluctuations after eating WR because the study participants were selected based on the results of a 75 g oral glucose tolerance test. Additionally, although we avoided setting experimental days during menstruation, there was no consideration of the menstrual cycle in that study [4]. It has been suggested that the menstrual cycle affects postprandial blood glucose levels [12]. Regarding women participants, we should only have considered postmenopausal women who are not affected by the menstrual cycle. Thus, additional studies are needed to confirm the effects of HPGA natto on inhibiting postprandial glucose elevation in humans with consideration of the results and limitations of our previous study. If the effects on the health of HPGA natto can be clarified, it may be possible to prevent lifestyle-related diseases by utilizing HPGA natto.

Therefore, in this study, we evaluated the suppressive effects of HPGA natto on postprandial glucose elevation with changes in the number and characteristics of the study participants relative to those in the previous study and focused on the IAUC for blood glucose 0 to 30 min after loading between the LPGA and HPGA meals.

## 2. Materials and Methods

### 2.1. Participants

Recruitment of participants was performed by the staff of the research team at the University of Tsukuba (Tsukuba, Ibaraki, Japan) through a newspaper ad and our study website. Prior to the screening test, we obtained written informed consent from all individuals.

In this study, eligibility of participants was assessed in two steps: the first screening test was the primary registration, and the second screening test was the secondary registration. The inclusion criteria in the primary registration were as follows: men aged 20 to 70 years and postmenopausal women aged less than 70 years; body mass index (BMI) from 18.5 to 24.9 kg/m^2^; fasting plasma glucose <126 mg/dL; hemoglobin A1c (HbA1c) <6.5%; the ability to maintain usual diets and lifestyles during the study period. Exclusion criteria were patients with diabetes and/or anemia, those taking medication for chronic illness, routinely taking supplements or health food that might affect the outcome of the study, those who were taking part in other clinical trials, and those who may experience an allergy related to the test meal.

**Figure 1:**
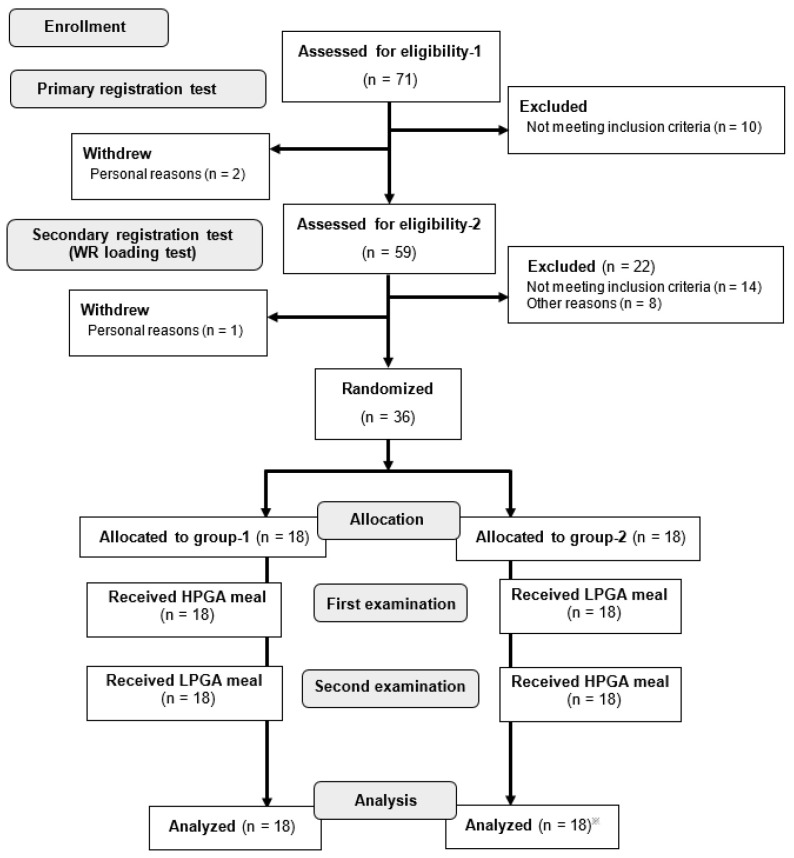
Study flowchart. Of the 71 candidates identified, 36 eligible individuals were randomly assigned to two groups. ※ All data for the second examination of one participant in group-2 were treated as missing data. LPGA: low-gamma-polyglutamic acid; HPGA: high-gamma-polyglutamic acid.

Next, a loading test with 150 g of packaged rice and 150 g of water as the secondary registration test (WR loading test) was performed in participants who were confirmed to be eligible for primary registration (*n* = 61). In the secondary registration, individuals whose postprandial glucose levels exceeded 200 mg/dL at one or more time points were excluded, and those with a blood glucose level ≥140 mg/dL at any time point were registered preferentially. In particular, disease-free volunteers with relatively elevated postprandial glucose levels among the participants of the secondary registration test were selected. These criteria were set based on the American Diabetes Association′s cut-off points [13]. In our previous study, the difference in the effects of the two types of natto may have been reduced because participants with low blood glucose elevation after WR eating were included [4]. Therefore, this secondary registration test was added in the present study to detect individuals who were not diabetic but had a relatively large elevation in blood glucose after WR loading, prior to undergoing two types of test meal loading tests. Of the 37 eligible participants, 36 were enrolled in the study and one person declined. We randomly divided the participants into two groups of 18. At the first examination, group-1 and -2 received the HPGA and LPGA meals, respectively. The type of natto included in the test meals was changed after the first meal and provided to each group at the next examination. One of the group-2 participants withdrew from the second examination for personal reasons (Figure 1).

The protocol of the present study was in accordance with the Declaration of Helsinki and was approved by the Clinical Research Ethics Committee of the University of Tsukuba Hospital (approval number: R01-116). This study was registered with the University Hospital Medical Information Network Clinical Trials Registry System (http://www.umin.ac.jp/ctr) as UMIN 000037449. Subsequently, data were collected between 18 August and 27 November 2019 at the University of Tsukuba by the staff of the research team.

### 2.2. Study Protocol

A single-blinded, randomized controlled cross-over study was conducted. A simple randomization method was used by the data coordinator, using a random number table generated by computer. The participants were blinded to their treatment allocation, but some of the research staff who were in charge of providing test meals to the participants could not be blinded.

The eligibility of participants was confirmed by primary and secondary registration tests.

A total of three meal loading tests, including a secondary registration test, were performed at least four days apart. On the day before each meal loading test, all participants were provided a standardized dinner, the composition of which was based on a report by Taniguchi-Fukatsu et al. [14]. Each meal loading test was performed in the morning after 12 h of overnight fasting. In each examination, blood samples were obtained after fasting and for 120 min after loading for measurement of the levels of blood glucose and serum insulin. Next, the IAUCs for blood glucose and insulin were calculated using the trapezoidal method [15]. The primary outcome was the difference in blood glucose IAUC 0–30 min after the meal between the LPGA and HPGA meals, based on the results of the exploratory study that we conducted previously [4]. Measures of the IAUC of the blood glucose and serum insulin levels at 0–15, 0–45, 0–60, 0–90, and 0–120 min, and the IAUC of the serum insulin levels at 0–30 min were the secondary outcomes.

The energy and the amount of protein, fat, and carbohydrate of the WR were the same as a previous study [4]. The composition of HPGA and LPGA natto per 40 g, which was the amount in each test meal, is shown in Table 1. The amount of *γ*-PGA of the LPGA and HPGA natto per 40 g was 57.6 ± 8.4 mg and 439.6 ± 15.1 mg, respectively.

Participants consumed each test meal in 10 min. Packaged rice (Sato Foods Co., Ltd., Niigata, Japan) was microwaved for 2 min and LPGA or HPGA natto (Natto Research Center of Takano Foods Co., Ltd., Omitama, Ibaraki, Japan) were divided into 8 equal parts and served to participants. The *γ*-PGA in both LPGA and HPGA natto was produced during the fermentation process, and these amounts were affected by the type of *Bacillus subtilis*.

### 2.3. Clinical Measurements

Participants wore light clothing and no shoes during the measurements of body weight and height. BMI was calculated as body weight (kg)/height squared (m^2^). These metrics were collected at the primary registration test.

### 2.4. Blood Sample Analysis

With a JCA-BM9130 analyzer (JEOL, Tokyo, Japan), the levels of blood glucose and HbA1c were measured by the glucose dehydrogenase and latex coagulation methods, respectively. For measurement of insulin levels, a chemiluminescent immunoassay by Roche Modular Analytics E170 (Roche Diagnostics GmbH, Mannheim, Germany) was used. The homeostasis model assessment of insulin resistance index was calculated using the formula proposed by Matthews et al. [16]. These analyses were conducted by Kotobiken Medical Laboratories, Inc. (Tsukuba, Ibaraki, Japan).

### 2.5. Data Analysis

The calculation of the sample size was based on the results of our exploratory study [4]. In that study, the mean and standard deviation (SD) of the difference of the blood glucose IAUC 0–30 min after the meal between LPGA and HPGA meals were estimated as 115.2 and 220.5 mg/dL, respectively. Under these assumptions, the minimum sample size such that the power of the primary analysis exceeds 80% was estimated as 31. In consideration of the information loss due to dropouts, the sample size was set to 36 participants.

Linear mixed model analyses were used to compare the IAUC values of blood glucose and serum insulin, and changes in the amount of blood glucose and serum insulin from 0 min to the peak point among the test meals where the blood glucose IAUC at each point was included as a dependent variable, the participants as a random effect, and the test meals (for primary analysis: LPGA and HPGA meals; for complementary analysis: LPGA and HPGA meals, and WR) and groups as fixed effects. The primary analysis was the t-test for the differences in the means between LPGA and HPGA meal estimated with the linear mixed model for the outcome of the blood glucose IAUC 0–30 min after the meal. Insulin levels at time points when hemolysis was observed in the serum samples were treated as missing data considering the deviation from the true value. In addition, for the one participant who could not participate on the third day of measurement, blood glucose and insulin levels on that day were also treated as missing data. These data are presented as the estimated marginal mean ± standard error (est. mean ± SE). Other descriptive data are shown as the mean ± SD if not specified. All statistical analyses were conducted with SPSS Statistics 25 software (SPSS, Inc., Chicago, IL, USA), and statistical significance was set to two-tailed *p* values of less than 0.05.

## 3. Results

### 3.1. Baseline Characteristics

As shown in Table 2, 36 participants (*n* = 18 in each group) who had participated in at least one of the two meal loading tests including natto were evaluated. Men accounted for 80.6% of the participants while women made up only 19.4% of our population.

At the primary registration test, 19.4% of subjects with prediabetes met the American Diabetes Association′s criteria [9]. In the secondary registration test, the blood glucose levels at the peak and 2 h after WR loading were 144.1 ± 19.2 (mean ± SD) mg/dL and 100.1 ± 13.5 mg/dL, respectively. There was no participant with blood glucose levels ≥140 mg/dL after the 2-h WR loading.

### 3.2. Comparison of Blood Glucose IAUC between LPGA and HPGA Meals

**Figure 2:**
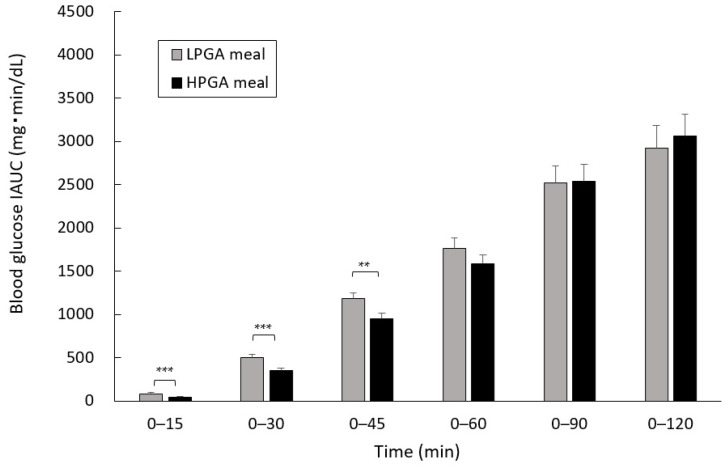
Effects of LPGA and HPGA meals on the levels of blood glucose IAUC. Liner mixed model analysis was used to compare the blood glucose IAUC at each time point, with the test meals (LPGA and HPGA meals) and groups as fixed effects. These data are presented as the est. mean ± SE. ** *p* < 0.01, *** *p* < 0.001. LPGA: low-gamma-polyglutamic acid; HPGA: high-gamma-polyglutamic acid; IAUC: incremental area under the curve.

The levels of blood glucose IAUC at 0 to 30 min, the primary outcome of the present study, of the HPGA meal (351.2 ± 28.0 (est. mean ± SE) mg·min/dL) were significantly lower than those generated by the LPGA meal (505.3 ± 36.0 mg·min/dL) (*p* < 0.001). The HPGA meal also showed significantly lower blood glucose IAUC than the LPGA meal at 0 to 15 and 0 to 45 min (0 to 15 min: *p* < 0.001, 0 to 45 min: *p* < 0.01) (Figure 2).

The results of the complementary analysis of the blood glucose IAUC of the WR were as follows: 0 to 15 min: 95.6 ± 10.6 (est. mean ± SE), 0 to 30 min: 540.2 ± 33.7, 0 to 45 min: 1318.7 ± 59.3, 0 to 60 min: 2124.3 ± 90.0, 0 to 90 min: 3354.7 ± 167.7, 0 to 120 min: 3994.3 ± 227.7 mg min/dL. No significant difference was observed between the blood glucose IAUC of the LPGA meal and WR at 0 to 15 and 0 to 45 min, whereas the blood glucose IAUC of both the LPGA meal (0 to 60 min: *p* < 0.01, 0 to 90 and 0 to 120 min: *p* < 0.001) and HPGA meal (all *p* < 0.001) at 0 to 60 and 0 to 120 min was significantly lower than those generated by WR.

The blood glucose levels of both the LPGA and HPGA meals peaked at 45 min. Although there was no significant difference, the increases in blood glucose levels at 0 to 45 min were in the order of the LPGA meal (45.4 ± 3.4 (est. mean ± SE) mg/dL), and the HPGA meal (43.2 ± 3.6 mg/dL). When the changing amount of blood glucose levels at 0 to 45 min of the WR were added to the linear mixed model, significant differences between the WR and the LPGA and HPGA meals were observed (LPGA meal: *p* < 0.01, HPGA meal: *p* < 0.001).

### 3.3. Comparison of Blood Glucose IAUC Considering the Degree of Blood Glucose Increasing after WR Eating

The blood glucose IAUC among test meals was also compared considering the degree of blood glucose elevation after WR eating. In particular, the individuals were divided into the low-glucose response group (*n* = 17) and high-glucose response group (*n* = 19) based on the average cut-off value of blood glucose IAUC after WR loading at 0 to 120 min (=3994.3 mg·min/dL).

**Figure 3:**
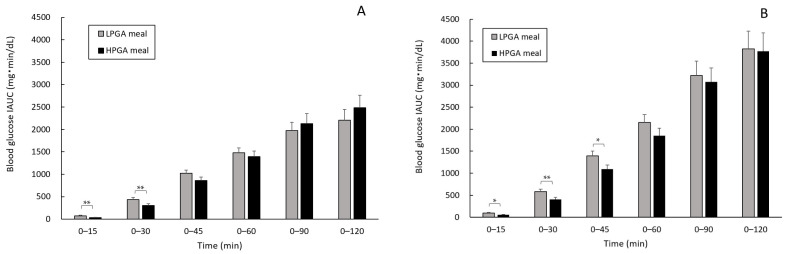
Effects of LPGA and HPGA meals on the levels of blood glucose IAUC in the low-glucose response group (**A**) and high-glucose response group (**B**). Linear mixed model analysis was used to compare the blood glucose IAUCs at each time point with the test meals (LPGA and HPGA meals) and groups as fixed effects, and these data are presented as the est. mean ± SE. *p < 0.05, **p < 0.01. LPGA: low-gamma-polyglutamic acid; HPGA meal: high-gamma-polyglutamic acid; IAUC: incremental area under the curve.

The HPGA meal had significantly lower blood glucose IAUC than that of the LPGA meal in the low-glucose response group at 0 to 15 min and 0 to 30 min (both *p* < 0.01, Figure 3A), whereas in the high-response group this effect was observed at 0 to 15 min and 0 to 45 min (0 to 15 and 0 to 45 min: *p* < 0.05, 0 to 30 min: *p* < 0.01, Figure 3B). Additionally, in the high-glucose response group, the HPGA meal showed low blood glucose IAUC compared to the LPGA meal at all time points (Figure 3B).

### 3.4. Comparison of Serum Insulin IAUC between LPGA and HPGA Meals

**Figure 4:**
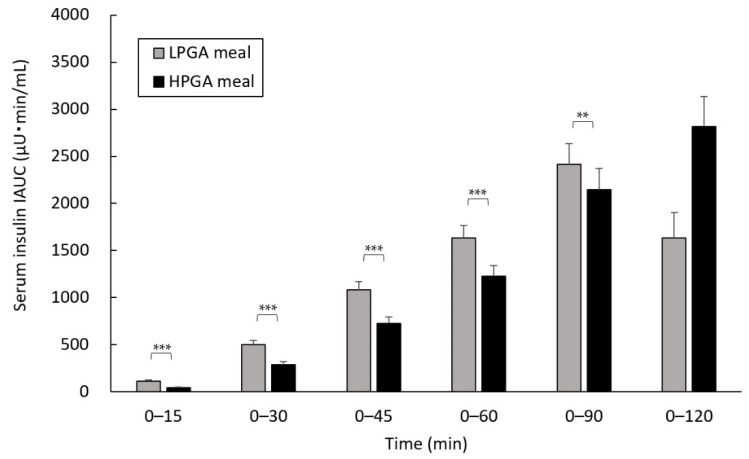
Effects of LPGA and HPGA meals on the levels of serum insulin IAUC. Linear mixed model analysis was used to compare the serum insulin IAUC at each time point, with the test meals (LPGA and HPGA meals) and groups as fixed effects. These data are presented as the est. mean ± SE. ** *p* < 0.01, *** *p* < 0.001. LPGA: low-gamma-polyglutamic acid; HPGA: high-gamma-polyglutamic acid; IAUC: incremental area under the curve.

The HPGA meal had lower serum insulin IAUC than the LPGA meal except at 0 to 120 min (at 0 to 15 and 0 to 60 min: *p* < 0.001, 0 to 90 min: *p* < 0.01) (Figure 4). The serum insulin secretion peaked at 45 min with the LPGA meal, whereas it was delayed to 60 min with the HPGA meal. The changes in serum insulin levels from 0 min to the peak point were in the order of LPGA meal (39.5 ± 4.0 (est. mean ± SE) mg/dL), HPGA meal (33.4 ± 3.6 mg/dL) (*p* = 0.070).

## 4. Discussion

In our study, the IAUCs for blood glucose and serum insulin of the HPGA meal were markedly lower than that of the LPGA meal within 45 min and 90 min after loading, respectively. This supports the findings of our previous exploratory study [4], and the suppressive effects of HPGA natto on blood glucose elevation in the postprandial initial phase were revealed. Although it was only the results of the complementary analysis, the blood glucose IAUC of the LPGA meal 1 to 2 h after loading and that of the HPGA meal within 2 h after loading were significantly lower than that of the WR. Thus, both LPGA and HPGA natto were considered effective for suppressing postprandial blood glucose elevation after WR eating, and HPGA natto was shown to be more effective than LPGA natto. The results of the stratified analysis, based on the average cut-off value of blood glucose IAUC after WR loading at 0 to 120 min, showed that the blood glucose IAUC of the HPGA meal was significantly lower than that of the LPGA meal at more time points in the high-glucose response group than in the low-glucose response group. Additionally, in the high-response group, the HPGA meal showed lower blood glucose IAUC than the LPGA meal at all time points. Therefore, the suppressive effects of HPGA natto for blood glucose elevation are probably more pronounced in participants who have a high glucose IAUC after WR eating. Based on the results that the blood glucose IAUCs at 0 to 60 min and 0 to 120 min were not different between LPGA and HPGA meals in the whole population, it was difficult to determine whether the improvement of postprandial glucose elevation by HPGA natto in the present study was clinically meaningful. However, considering the results in the high-response group, it is expected that the suppressive effect of HPGA natto for postprandial glucose elevation may be obtained at all time points if we targeted individuals with higher blood glucose levels.

In the animal study by Tamura et al., the inhibitory effect of *γ*-PGA on the blood glucose levels increased at the initial phase after starch loading [17]. *γ*-PGA is a viscous substance [3], and its content is approximately eight-fold higher in HPGA natto than in LPGA natto. β-glucan, a water-soluble dietary fiber, has high viscosity [6] like *γ*-PGA. β-glucan increases the viscosity of the food bolus and slows down the movements in the digestive tract, which delays gastric emptying and absorption [18]. The reduction of starch digestibility and the glucose responses were more pronounced depending on the increasing viscosity of β-glucan [19]. Compared to the LPGA meal, the HPGA meal had lower peaks of both blood glucose and serum insulin, and insulin peak was delayed in our study. The HPGA natto in our study may have suppressed the blood glucose elevation through a mechanism similar to that of β-glucan.

In our study, the carbohydrate, protein, and dietary fiber contents in both test meals were similar. Additionally, the blood glucose IAUC between the HPGA and LPGA meals did not differ 1 h after loading. However, the serum insulin IAUC of the HPGA meal was significantly lower than that of the LPGA meal at all time points, except 0 to 120 min. Thus, HPGA natto effectively suppressed postprandial glucose elevation with a small amount of insulin. This may be similar to the effect of acarbose, an oral anti-diabetic drug that delays starch digestibility and absorption via the reversible inhibition of *α*-glucosidase [20]. In addition, although there are no unified views [21,22,23], it has been reported that long-term acarbose treatments improve insulin sensitivity [22,23] and secretion [23]. The efficacy of *α*-glucosidase inhibitors for preventing or delaying the onset of type 2 diabetes had been suggested in individuals with impaired glucose tolerance (IGT) and those at an increased risk for diabetes [24]. Therefore, the HPGA natto may also contribute to the prevention of IGT and type 2 diabetes. This is important because mild hyperglycemia has been reported to cause pancreatic β-cell damage via alterations in gene expression [25].

Postprandial hyperglycemia is a risk factor for cardiovascular events [10,26], and blood glucose levels 2 h after meals are generally used to assess the risk of developing type 2 diabetes [13]. However, plasma glucose levels 1 h after loading have been demonstrated as a better predictor of dysglycemia than plasma glucose levels 2 h after loading [27]. A relationship has been reported between the 1 h post-load plasma glucose levels of middle-aged men with normal glucose tolerance and arterial stiffness [28]. Esposito et al. reported that 95% of 644 patients with type 2 diabetes had a peak blood glucose level within 1 h after eating and that elevated blood glucose levels at the peak correlated strongly with the CIMT [29]. In their other study, the mean peak blood glucose of type 2 diabetic patients was also within 1 h, and the regression of CIMT by decreasing the glucose peak and IAUC was observed [11]. Due to the suppression of blood glucose elevation within 45 min after meal consumption was greater in the case of HPGA meal compared to that in the case of LPGA natto, using HPGA for the prevention of cardiovascular disease is possible.

This study had some limitations. The mechanism underlying the effects of HPGA natto on postprandial blood glucose metabolism remains unclear. Additionally, this study was conducted on disease-free volunteers with relatively elevated postprandial glucose levels in a limited area. The ratio of men to women in our population was biased and there was insufficient evidence for extrapolating these findings to females. We could not determine whether the suppressive effect of HPGA natto for postprandial glucose elevation has a clinical significance from the results of this single-dose study. Therefore, further studies including verification of the long-term effects are needed to confirm the generalizability of our results.

## 5. Conclusions

In this study, the blood glucose IAUC of the HPGA meal was remarkably lower than that of the LPGA meal within 45 min after loading. In addition, the HPGA meal suppressed insulin secretion more than the LPGA meal. Our results revealed that HPGA natto suppresses blood glucose elevation with a small amount of insulin secretion in the early postprandial phase. If the long-term continuous eating effects of HPGA natto on preventing dysglycemia and cardiovascular disease will be revealed in further studies, lifestyle-related diseases may be prevented by utilizing the HPGA natto as a functional food.

## Figures and Tables

**Table 1:** Composition of the LPGA and HPGA natto per 40 g.

	LPGA Natto	HPGA Natto
Energy (kcal)	76	77
Protein (g)	7.2	7.1
Fat (g)	3.6	3.9
Carbohydrate (g)	5.1	5.1
Dietary fiber (g)	3.3	2.5
*γ*-PGA (mg)	57.6	439.6

LPGA: low-gamma-polyglutamic acid; HPGA: high-gamma-polyglutamic acid; *γ*-PGA: gamma-polyglutamic acid.

**Table 2:** Baseline Characteristics.

Characteristic	Value
Number	36
Men/women (%)	80.6/19.4
Age (years)	40.5 ± 16.4
Body mass index (kg/m^2^)	21.7 ± 1.7
Fasting plasma glucose (mg/dL)	89.6 ± 8.5
Hemoglobin A1c (%)	5.4 ± 0.2
Insulin (*µ*U/mL)	5.2 ± 2.4
Homeostasis model assessment of insulin resistance	1.2 ± 0.6
